# Supplementary material for: Empirical methods for controlling false positives and estimating confidence in ChIP-Seq peaks
Source: BMC Bioinformatics. 2008 Dec 5;9:523. doi: 10.1186/1471-2105-9-523 (PMC2628906; doi:10.1186/1471-2105-9-523)
Supplement: Additional File 1 — A variety of html documents from the USeq web site detailing the available applications, their best usage, output file type descriptions, command line menus, etc. [file 1471-2105-9-523-S1.zip › USeqUserGuides/JavaDoc.zip!/JavaDoc/trans/misc/ConvertGeoData.html]

ConvertGeoData


|  |  |  |
| --- | --- | --- |
| **Overview** \| **Package** \| **Class** \| **Use** \| **Tree** \| **Deprecated** \| **Index** \| **Help** | |  |
| **PREV CLASS**   **NEXT CLASS** | **FRAMES**    **NO FRAMES**     **All Classes** |  |
| SUMMARY: NESTED \| FIELD \| CONSTR \| METHOD | DETAIL: FIELD \| CONSTR \| METHOD |  |


---


## trans.misc Class ConvertGeoData

```
java.lang.Object
  trans.misc.ConvertGeoData
```

---

``` public class ConvertGeoData extends java.lang.Object ```

Reads and parses in a GEO data file into a tpmap and xxx.cela files for TiMAT2 processing. ie
#ID\_REF =
#Rval = Cy5 (red) signal, Input total DNA from WI38 cells
#Gval = Cy3 (green) signal, MeDIP DNA from WI38 cells
#VALUE = log2(Gval/Rval)
ID\_REF Rval Gval VALUE
CHR1P000009757\_HSAP0406S00000003 2934.44 6303.33 1.1030
CHR1P000009637\_HSAP0406S00000003 4742.78 13346.00 1.4926
CHR1P000009627\_HSAP0406S00000003 3311.56 8825.89 1.4142
CHR1P000009617\_HSAP0406S00000003 3295.78 8769.11 1.4118
CHR1P000009607\_HSAP0406S00000003 3812.56 10092.00 1.4044
Saves xxx.cela files, and a xxx.tpmap

---

**Constructor Summary**

`ConvertGeoData(java.lang.String[] args)`


| **Method Summary** | |
| --- | --- |
| `int` | `countDataLines(java.io.File file)` |
| `static void` | `main(java.lang.String[] args)` |
| `boolean` | `parseIt(java.io.File file)` |
| `static void` | `printDocs()` |
| `void` | `processArgs(java.lang.String[] args)`             This method will process each argument and assign new varibles |

**Methods inherited from class java.lang.Object**

`equals, getClass, hashCode, notify, notifyAll, toString, wait, wait, wait`

**Constructor Detail**

### ConvertGeoData

```
public ConvertGeoData(java.lang.String[] args)
```


**Method Detail**

### parseIt

```
public boolean parseIt(java.io.File file)
```

---


### countDataLines

```
public int countDataLines(java.io.File file)
```

---


### processArgs

```
public void processArgs(java.lang.String[] args)
```

:   This method will process each argument and assign new varibles

---


### printDocs

```
public static void printDocs()
```

---


### main

```
public static void main(java.lang.String[] args)
```


---


|  |  |  |
| --- | --- | --- |
| **Overview** \| **Package** \| **Class** \| **Use** \| **Tree** \| **Deprecated** \| **Index** \| **Help** | |  |
| **PREV CLASS**   **NEXT CLASS** | **FRAMES**    **NO FRAMES**     **All Classes** |  |
| SUMMARY: NESTED \| FIELD \| CONSTR \| METHOD | DETAIL: FIELD \| CONSTR \| METHOD |  |


---
